# Human pluripotent stem cell-derived brain organoids as in vitro models for studying neural disorders and cancer

**DOI:** 10.1186/s13578-021-00617-1

**Published:** 2021-05-28

**Authors:** Juan Luo, Peng Li

**Affiliations:** Scientific Research Center, The Seventh Affiliated Hospital of Sun Yat-Sen University, Shenzhen, 518107 China

**Keywords:** Human pluripotent stem cells, Brain organoids, Neural disorders, Cancer

## Abstract

The sheer complexities of brain and resource limitation of human brain tissue greatly hamper our understanding of the brain disorders and cancers. Recently developed three-dimensional (3D) brain organoids (BOs) are self-organized and spontaneously differentiated from human pluripotent stem cells (hPSCs) in vitro, which exhibit similar features with cell type diversity, structural organization, and functional connectivity as the developing human brain. Based on these characteristics, hPSC-derived BOs (hPDBOs) provide new opportunities to recapitulate the complicated processes during brain development, neurodegenerative disorders, and brain cancers in vitro. In this review, we will provide an overview of existing BO models and summarize the applications of this technology in modeling the neural disorders and cancers. Furthermore, we will discuss the challenges associated with their use as in vitro models for disease modeling and the potential future direction.

## Overview of hPDBOs

The complexity of human brain, including the cell type diversity, cellular architecture and functional connectivity, makes understanding its secrets to be one of the most fascinating things in science [1]. However, the difficulty in accessing of human brain tissues greatly limited our investigation and understanding to various of neurological disorders. Although some studies using the animal model provide valuable mechanistic insights into the pathogenesis and causes of human brain disorders, their values are limited because of the species differences. Therefore, it is urgent to develop an alternative experimental system to study human brain development and disorders, and which should be able to mimic the human context and ethically just.

hPSCs, including embryonic stem cells (ESCs) and induced pluripotent stem cells (iPSCs), can propagate in vitro and differentiate into all adult cells, they therefore hold great promise for disease remodeling and drug discovery [2–5]. Particularly, the iPSCs derived from patients substantially facilitated us to recapitulate some of the disease-associated phenotypes with brain disorders from the cellular level [6]. Combination of genome-editing technology with hPSC model enables scientists to recapitulate any disease-associated mutations in cultured cells, and further correct of these genetic mutations in patient-derived iPSCs can be used to validate these phenotypes [7–9]. These technologies indeed prove a boon for revealing cellular phenotypes associated with brain development and disorders [10]. However, most of the disease phenotypes are still difficult to be recapitulated using the monolayered culture system, owing to lack of the tissue architecture and microenvironment. To this end, the hPDBOs, which are self-organized in vitro and resemble the embryonic human brain, including cell type diversity and cytoarchitecture [11–14], provide a unique platform to investigate the complicated processes during brain development, neurodegenerative disorders and tumorigenesis [15, 16].

In 2008, Eiraku et al. firstly adopted a 3D aggregate culture system to differentiate ESCs into cortical progenitors, the resulting embryoid body (EB)-like aggregates exhibited regional dorsal–ventral specification and recapitulated the early aspect of corticogenesis [17, 18]. In 2013, Lancaster et al. modified this protocol by introducing Matrigel droplets and Spinning bioreactor to maintain 3D structure of the aggregates and continuous supplies of oxygen and nutrients, the resulting aggregates (called BOs) displayed structural features like human brain, such as establishing the apical-basal polarity and expressing region-specific markers within the cortical domain, including forebrain, midbrain, hindbrain, hippocampus etc. [11, 19, 20]. Subsequently, by extending the culture time with an improved Spinning mini-bioreactor or a sliced neocortical organoid (SNO) system, an expanding cortical plate was developed, which constitutes neural subtypes of all six cortical layers and resembles with the third trimester embryonic human neocortex [21, 22]. Transcriptome comparisons of human BOs and fetal neocortex at different stages by scRNA-seq showed the organoids also closely recapitulated the development of glial lineage cells, and their gene expression patterns were reminiscent of fetal human brain development [12, 14, 23–26]. More importantly, when the in vitro-developed human BOs were transplanted to adult mouse brain, they showed progressive neural differentiation, maturation, and established the graft-to-host functional synaptic connectivity, which facilitated us to model brain diseases under physiological conditions [27].

Up to now, various types of defined protocols have been developed to differentiate hPSCs to region-specific BOs, including forebrain, midbrain, hindbrain, hippocampus, choroid plexus, hypothalamus, and cerebellum organoids, all of them have recapitulated the molecular, cellular, and structural features of the corresponding areas of human brain [21, 28–32]. Relying on these technologies, the researchers have developed the “assembloids” by fusing differentially patterned region-specific BOs, such as forebrain dorsal and ventral fusion, and forebrain fusion with thalamus [33–38]. These assembloids enable us to explore the complex interactions between different parts of brain, and will shed light on more complicated processes of brain development and diseases. In 2019, Giandomenico et al. developed an air–liquid interface cerebral organoid (ALI-CO) system, and by culturing with mouse spinal cord sections, the resulting organoids have established synapses with spinal cord neurons, which could further guide mouse muscle contraction [39]. Recently, Andersen et al. have assembled BOs and spinal cord-like organoids, together with human skeletal muscle spheroids to generate the cortico-motor assembloids. In this model system, they observed the corticofugal neurons can project and connect with spinal spheroids to control muscle contraction [40]. These studies highlighted the remarkable self-assembly capacity of 3D cultures to form a functional circuit, and which could be used to understand previously inaccessible features of human brain function, such as cell–cell interactions and neural circuit formation in whole central nervous system (CNS) [41–43].

**Fig. 1:**
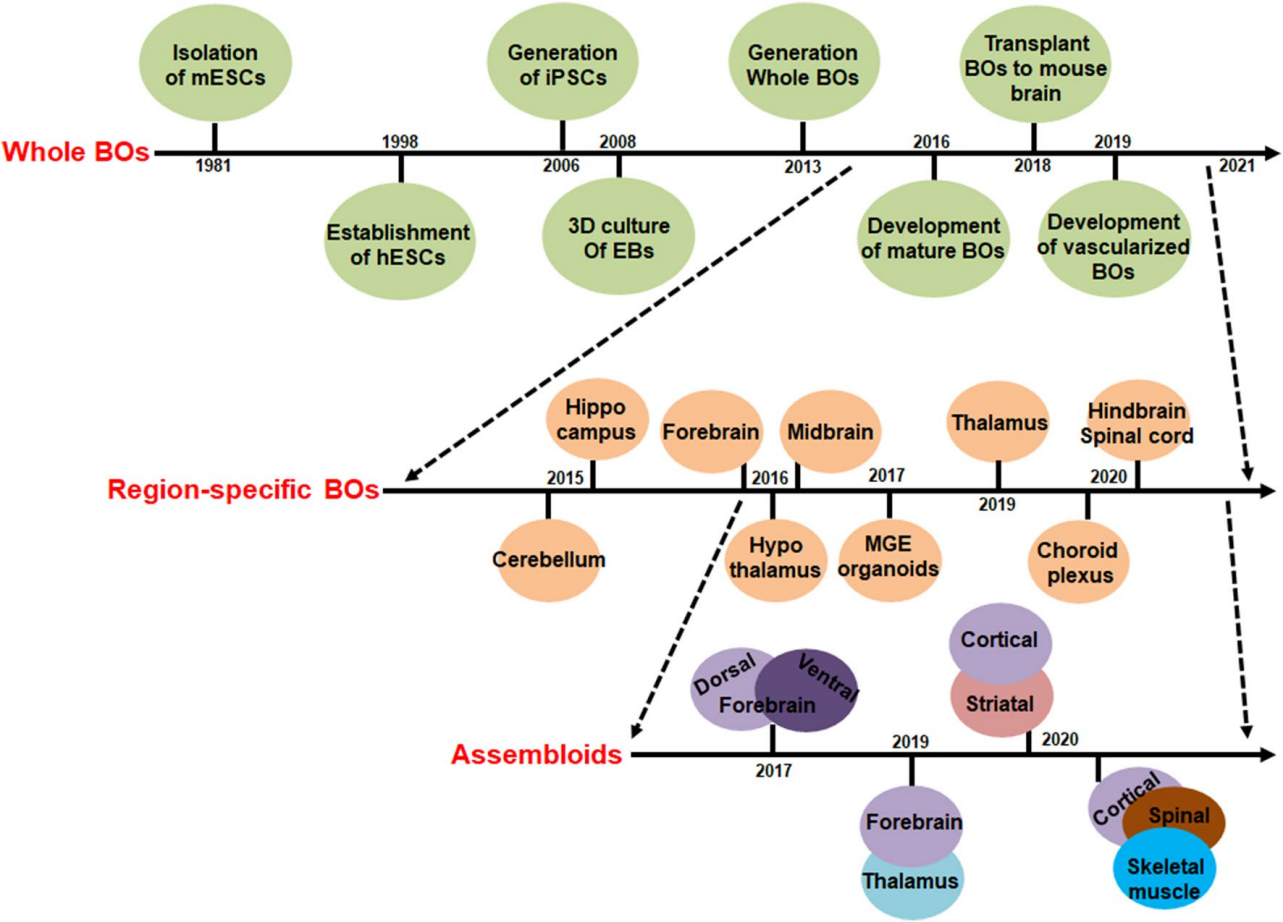
Development of brain organoid technologies: Timeline cartoons to indicate the milestones from isolation of m/hESCs to establishment of mature hPDBOs, including vascularized BOs, region-specific BOs and assembloids

In the last decade, the hPDBO model, including the whole brain, region-specific BOs and assembloids (Fig. 1 and Table 1), has been widely used to investigate human brain disorders and cancers (Table 2). In this review, we summarize the current applications of hPDPOs in these aspects, and will discuss the challenges of their use as in vitro models and the potential future directions.

**Table 1:** Types of hPSC-derived brain organoids

Organoid type	Brain region	Methodology	Culture conditions	Observed phenotypes	Main reference
EB-like aggregates	Cerebral cortex	Guided	Stationary floating culture, followed by re-plating of aggregates	The cortical tissues contained ventricular, early and late cortical-plate, and Cajal-Retzius cell zones along the apico-basal direction	[18]
Cerebral organoid	Whole brain	Unguided	Matrigel and spinning bioreactor	Apical-basal polarity, interkinetic nuclear migration, division modes of neural stem cells, and the pattern of neuronal migration were well maintained, and the enlarged OSVZ was exhibited	[11]
			Matrigel and spinning bioreactor	The molecular map of the diversity of cell types across organoids is provided by sc-RNA sequencing, and they showed organoids undergo substantial neuronal maturation	[12]
			Intracerebral grafting of brain organoids into mouse brain	The organoids showed progressive neuronal differentiation and maturation, gliogenesis, and established the graft-to-host functional synaptic connectivity	[27]
Region-specific brain organoid	Cerebellum	Guided	Stationary floating culture	The induced cells self-organized into neural-tube-like structures with dorso-ventral and apico-basal polarities, and eventually formed layered structures that recapitulate cerebellar ontogenesis	[30]
	Forebrain		Matrigel and miniaturized multi-well spinning bioreactor	Forebrain organoids exhibited multi-layer progenitor zone organization and generated diverse neuronal subtypes of all six cortical layers	[21, 22]
	Midbrain		Matrigel and miniaturized multi-well spinning bioreactor	Midbrain organoids showed organized neuroepithelium-like structures expressing NESTIN and floor-plate precursor marker FOXA2 at early stage, TH^+^ DA neurons, NURR1^+^ and PITX3^+^ cells appeared at late stage	[21, 22]
	Hypothalamus		Matrigel and miniaturized multi-well spinning bioreactor	Hypothalamus organoids expressed NKX2.1, SOX2, NESTIN and FOXA2 at early stage, and then the peptidergic neuronal markers and homeobox protein OTP appeared at late stage	[21, 22]
	Hippocampus		Floating culture permeable film-based culture plates, mechanical cutting	The organoids produced Zbtb20^+^/Prox1^+^ granule neurons and Zbtb20^+^/KA1^+^ pyramidal neurons, and both of which were electrically functional with network formation	[31]
	Choroid plexus		Floating culture in 40% oxygen	The organoids produced Lmx1a^+^, Otx2^+^ and TTR^+^ neurons, which are neuronal markers for choroid plexus	[31, 32]
	MGE organoid		Orbital shaker	The organoids specifically enriched the NKX2-1^+^ and DLX2^+^ neurons like in MGE	[33]
	Thalamus		Orbital shaker	The organoids specifically enriched the OTX2^+^, GBX2^+^ and DBX1^+^ neurons like in thalamus	[34]
	Hindbrain/spinal cord		Stationary floating culture	The neurons in hindbrain/spinal cord have established synaptical connection with neurons in cortical organoids	[40]
Fused organoids: “assembloids”	Dorsal and ventral forebrain	Guided	Stationary floating culture	The assembloids have recapitulated the interactions between glutamatergic neurons and GABAergic interneurons resembling the dorsal and ventral forebrains in human brain	[37]
	Dorsal and ventral forebrain		Orbital shaker	The assembloids have recapitulated the interactions between glutamatergic neurons and GABAergic interneurons resembling the dorsal and ventral forebrains in human brain	[38]
	Thalamus and forebrain		Orbital shaker	The axons from both cortex and thalamus reach the other side within 6 days in assembloids	[34]
	Cortical-mouse spinal cord		Air–liquid interface cerebral organoid method	Synapses could be detected between ALI-CO projecting axons and spinal cord neurons; axon tracts could guide mouse muscle contraction when innervated	[39]
	Cortico-striatal assembloids		Stationary floating culture	The cortical neurons send axonal projections into striatal organoids and form synaptic connections in assembloids	[35]
	Cortico-motor assembloids		Stationary floating culture	The corticofugal neurons could project and connect with spinal spheroids, while spinal-derived motor neurons further connect with muscle to control its contraction	[40]

**Table 2:** hPSC-derived brain organoids for modelling neural disorders and cancer

Disease	Genes/Cell types	Brain region/Organoid type	Observed phenotypes in organoids	Conclusions	Reference
Microcephaly	*CDK5RAP2*/ Patient iPSC	Whole brain/cereal organoid	Patient organoids showed a smaller overall size and a reduction of the progenitor zones	*CDK5RAP2* mutation in brain organoids leads to decreased NPCs proliferation and premature NPCs differentiation	[11]
	*CPAP*/ Patient iPSC		Patient organoids exhibited a reduced size, enlarged ventricular lumen, increased number of cilia as well as premature neurogenesis	*CPAP* mutation causes cilia disassembly, and eventually leads to a delayed cell-cycle re-entry and premature neurogenesis	[51]
	*WDR62*/Genetically modified iPSC		*WDR62* depletion resulted in smaller cerebral organoid sizes due to reducing the oRGs proliferation and inducing the early neurogenesis	WDR62-CEP170-KIF2A pathway functions as a contributor to microcephaly	[59]
	*NARS1*/Patient iPSC		Patient organoids showed reduced proliferation of radial glial cells, resulting in smaller organoids characteristic of microcephaly	NARS1 is required to meet protein synthetic needs and to support RGC proliferation in human brain development	[60]
	*IER3IP1*/Genetically modified iPSC		*IER3IP1* KO organoids showed a smaller size, neural rosettes and reduced NPCs compared to WT organoids	The regulation of extracellular matrix protein secretion by IER3IP1 is involved in brain size control and is implicated in microcephaly	[61]
Epilepsy	*TSC1/2*/Genetically modified iPSC	Whole brain/cereal organoid	Loss of *TSC1* or *TSC2* in brain organoids prominently disrupted the developmental suppression of mTORC1 pathway and resulted in abnormal differentiation and hypertrophy of human neurons and glia, giving rise to dysplastic cells resembling those found in tubers	mTOR inhibition during a critical developmental period prevents the formation of dysplastic cells	[79]
	*CACNA1C*/Timothy syndrome patient iPSC	Cortical-subpallium/ Assembloids	Abnormal salutatory migration of interneurons was observed in patient assembloids with TS	Treating the assembloids with L-type calcium channels inhibitors (nimodipine) can rescued the saltatory defects	[37]
Autism spectrum disease (ASD)	*FOXG1*/ASD patient iPSC	Whole brain/cereal organoid	ASD-derived organoids exhibited an accelerated cell cycle and overproduction of GABAergic inhibitory neurons	Overexpression of *FOXG1* induced GABAergic neuron fate is a developmental precursor of ASD	[83]
	*CDH8*/Genetically modified iPSC		Transcriptome analysis using cerebral organoids with CHD8 haplosufficiency revealed a subset of dysregulated genes overlapping those of the idiopathic ASD organoids	CHD8 may be a genetic risk of ASD	[84]
	*RAB39b*/Genetically modified iPSC		Brain organoids carrying the *RAB39b* gene mutation showed enlarged sizes	*RAB39b* mutation promotes PI3K-AKT-mTOR activity and alters cortical neurogenesis, leading to ASD-like behaviors	[87]
Alzheimer’s disease (AD)	*APP* and *PSEN1*/AD patient iPSC	Whole brain/cereal organoid	AD-associated brain organoids showed increased amyloid aggregation, hyperphosphorylated tau protein, and increased apoptosis	Brain organoids represent an alternative model for studying the pathology of genetic forms of AD	[95]
	*APOE4*/AD patient iPSC		AD patients carrying APOE4 increased levels of Aβ and phosphorylated tau, and exacerbated neuronal cell apoptosis and synapse loss	APOE4 exacerbates AD pathogenesis in cerebral organoids	[97]
	*PITRM1*/Genetically modified iPSC		*PITRM1* knockout organoids showed accumulation of protein aggregates, tau pathology, and neuronal cell death	PITRM1 plays a protective role of in AD pathogenesis	[98]
	*BACE2*/AD patient iPSC		Trisomy of *BACE2* skewed non-amyloidogenic Aβ peptide ratios and suppresses AD-like pathology in organoids	BACE2 is an AD suppressor in human brain	[102]
Parkinson’s disease (PD)	*LRRK2*^*G2019S*^ /PD patient and genetically modified iPSCs	Midbrain/midbrain organoid	*LRRK2*^*G2019S*^ hiPSC-derived midbrain organoids showed reduction of the number and arborisation complexity of TH^+^ cells, impaired mitochondrial function, and increased apoptosis	LRRK inhibition and knockdown of *TXNIP* can rescue the observed phenotypes in PD midbrain organoids	[110, 111]
Huntington’s disease (HD)	*HTT*/ integration-free HD hiPSC with 21, 28, 33, 60, 109, and 180 CAG repeats	Whole brain/cereal organoid	HD hiPSC-derived organoids showed immature ventricular zone and defects in neuroectoderm and rosette formation	HD-associated phenotypes, including the cortical and striatal specification defects, and neuronal migration and differentiation defects are CAG expansion dependant	[114]
GBM initiation	*MYC*^*OE*^, *NF1*^*−*^*/PTEN*^*−*^*/TP53*^*−*^, and *EGFRvIII*^*OE*^*/CDKN2A*^*−*^*/PTEN*^*−*^*/*genetically modified iPSC	Whole brain/neoCOR	Genetically modified cerebral organoids induced overgrowth and exhibited many features of cancer	neoCOR is an ideal model to study GBM initiation and progression in human brain-like tissues	[122]
	*HRas*^*G*12*V*^*/TP53*^*−*^*/*genetically modified iPSC		Genetically modified cerebral organoids induced mesenchymal GBMs and exhibited an invasive phenotype orthotopically xenografted into immunodeficient mice		[123]
GBM invasion		Co-culturing of GBM spheroids with cerebral organoids	Tumor spheroids could spontaneously infiltrate early-stage cerebral organoids and exhibited an invasive phenotype	Co-culture model is an ideal to study GBM/GSCs growth and invasive behaviours in brain-like tissues	[124]
		Co-culturing of GSCs with cerebral organoids: GLICO (GLIoma cerebral organoids)	GSCs home toward the human cerebral organoid and deeply invaded and proliferated within the host tissue		[125]

## BOs for modeling neurodevelopmental disorders

Neurodevelopmental disorders are the diseases that impair brain functions such as emotions, learning, sociality, or self-control due to perturbations in the developmental processes. Microcephaly, Epilepsy, and Autism spectrum disorder (ASD) are well known examples [44]. More than 5% of the world population is suffering from neurodevelopmental disorders [45]. However, the pathological causes of them are largely unknown due to lack of a proper preclinical model by which to study the disease mechanisms and develop new treatments. hPDBOs are expected to provide new breakthroughs in this field, owing to the similarities of BOs and human brain in origin and development processes. In this section, we summarize recent findings using hPDBOs for neurodevelopmental disease modeling and drug screening.

### Modeling microcephaly with BOs

Microcephaly is a typical neurodevelopmental disorder characterized by markedly reduced brain size, and the thin and not well layered cortical layers in patients [46]. Microcephaly is classified as congenital and acquired in terms of the cause of this disease, and lots of mouse models have been used to study the pathogenesis of genetic microcephaly. However, they were unable to closely recapitulate the microcephaly-associated phenotypes like in human patients, due to lack of the outer radial glia cells (oRGs) in human brain, which have been identified to be responsible for differentiation of the majority of upper-layer neurons [47–49]. hPDBOs have been demonstrated to contain these specific neural oRGs, and are eminently suitable for microcephaly modeling and drug screening [12, 25, 50]. So far, three independent centrosome-related BOs have been generated from microcephaly patient iPSCs carrying mutations in *CDK5RAP2*, *CPAP* and *ASPM* [11, 51, 52]. CDK5RAP2 is a pericentriolar material protein (PCM) in a centrosome and its mutations will lead to aberrant centrosomes function [53–55]. In 2013, Lancaster et al. firstly generated the patient iPSC-derived BOs carrying a compound heterozygous nonsense mutation in *CDK5RAP2*, and these organoids present a smaller overall size and reduction of the progenitor zones compared to controls, because of the abnormal proliferation and differentiation of oRGs in patient organoids [11]. In addition, as a CDK5RAP2-interacting protein, CPAP is a centriole wall protein required to assemble and recruit PCM proteins to a developing centrosome [56, 57]. Despite several independent *CPAP* mutations have been identified in microcephaly patients, the underlying mechanism is unknown. In 2016, Gabriel et al. generated stable iPSCs from microcephaly patient-derived fibroblasts with a deletion of exons 11–13 in *CPAP*. These patient iPSC-derived BOs exhibit a reduced size, enlarged ventricular lumen, increased number of cilia as well as premature neurogenesis, owing that the mutant CPAP protein caused cilia disassembly, and eventually lead to a delayed cell-cycle re-entry and premature neurogenesis [51]. These results suggested that maintaining the normal centrosome function play a critical role in expanding human neural progenitor cells (NPCs) and avoiding of microcephaly. Mutations in *WDR62* are the second most common genetic cause of autosomal recessive primary microcephaly in human [58]. Using the *WDR62*-deficient hPDBOs, Zhang et al. showed that *WDR62* depletion resulted in smaller BO sizes due to reducing the oRGs proliferation and inducing the early neurogenesis. On a molecular level they identified a novel WDR62-CEP170-KIF2A pathway as a contributor to microcephaly [59]. These studies demonstrated the hPDBOs provide a reliable platform to recapitulate the microcephaly-associated phenotypes and identify the molecular causes of microcephaly. Additionally, this model can be used to screen new microcephaly-associated genes. Wang et al. identified biallelic missense and frameshift mutations in *NARS1* in seven patients from three unrelated families with microcephaly, and these patient-specific BOs showed reduced proliferation of RGCs, resulting in smaller organoid characteristics of microcephaly, suggesting that NARS1 is required to support RGC proliferation in human brain development [60]. Recently, Esk et al. performed a loss-of-function (LOF) screen by CRISPR-LIneage tracing at cellular resolution in human BO tissue, and they tested 173 microcephaly candidate genes and identified 25 of them to be involved in known and uncharacterized microcephaly-associated pathways [61]. In summary, hPDBOs facilitated us to identify new microcephaly genes, and mechanisms involved in brain size control and drug screen in the future.

Besides the inherited microcephaly, Zika virus (ZIKV) pandemic in the Americas has demonstrated to be able to cause microcephaly [21, 62–64]. BOs exposed to ZIKV at different developmental stages showed ZIKV could directly target NPCs at the ventricular zones and cause depletion of NPCs, leading to the overall size reduction of organoids as seen with genetically inherited primary microcephaly [65, 66]. Subsequently, using the human forebrain organoid model, they further revealed that ZIKA-NS2A reduced RGC proliferation by impairing adherens junction complex formation and aberrant radial glial fiber scaffolding [67]. Moreover, several drug screens followed with validation using BO model have identified compounds such as emricasan, hippeastrine, 25-hydroxycholesterol and certain antibiotics and antivirals as promising candidates for treating Zika virus infection [68–71].

### Modeling epilepsy with BOs

Epilepsy is one of the most common neurological disorders and characterized by recurrent and unprovoked seizures due to neuronal hyperactivity. Epilepsy is etiologically heterogenous and antiepileptic drugs are the primary treating method. However, approximately 40% of patients have medically refractory seizures [72, 73]. So far, more than 500 loci of gene mutations are identified to be associated with epilepsy, but the molecular mechanisms remain largely unknown, even though they ultimately lead to brain hyperexcitability [74]. For genetic epilepsy, understanding the pathological mechanism is critical in identifying innovative therapeutic approaches. hPDBOs, with its more complex spatial structure, cell diversity and mature neural networks, have been widely used for modeling epilepsy-related disorders. Tuberous sclerosis complex (TSC) is an autosomal dominant congenital disorder characterized by growing benign tumors in multiple organs [75, 76]. Tumors presenting in brain, accumulating lots of enlarged and dysplastic neurons and glias in the cortex, are associated with epilepsy [77]. The LOF mutations of either *TSC1* or *TSC2* genes, which encode hamartin and tuberin respectively, will lead to the cortical tubers in brain due to hyperactivation of mTOR pathway that causes aberrant cell growth [78]. Blair et al. have generated hPDBOs carrying the null allele of *TSC1* or *TSC2* by gene-editing technology, and found homozygous loss of *TSC1* or *TSC2* exhibited the phenotypes resembling those found in tubers [79]. Timothy syndrome (TS) is another neurodevelopmental disorder characterized by epilepsy [80]. Birey et al. developed an in vitro assembloid by fusing human cortical spheroid (hCS) and subpallium spheroid (hSS) derived from TS patients. These hCS-hSS organoids showed an increase in saltation frequency, as well as a decrease in saltation length and speed, and which could be rescued by treating the assembloids with L-type calcium channels inhibitors, suggesting LTCC blockers might be used to prevent migration defects in patients with TS [37]. Undoubtedly, these studies demonstrated that hPDBOs provided a reliable platform for genetic epilepsy modeling and drug screening.

### Modeling ASD with BOs

ASD is a neurodevelopmental disorder and characterized by the defects of social interaction and communication, as well as repetitive behaviors. ASD is involved in the cell–cell interactions of many different cell types in brain, which makes it difficult to model with traditional 2D cell culture. Its pathophysiology remains elusive [81, 82]. The emergence of hPDBOs, in particular the assebmloids, holds this potential, as they can establish discrete regions of the brain [12, 14, 23, 24]. So far, some of the ASD-associated mechanisms have been identified using these models. In 2015, Mariani et al. generated BOs using the patient iPSCs with idiopathic ASD and demonstrated these organoids exhibit an accelerated cell cycle and overproduction of GABAergic inhibitory neurons, due to the overexpression of transcription factor *FOXG1*, revealing that FOXG1-induced GABAergic neuron fate is a developmental precursor of ASD [83]. *CHD8*, one of the most frequently mutated genes in ASD, encodes a member of ATP-dependent chromatin-remodeling factors [84]. The iPSCs carrying the *CHD8*^+*/−*^ mutation have been used to generate the BOs and subjected to do RNA-sequencing analyses. The results showed that CDH8 regulates the expression of genes implicated ASD, such as *TCF4* and *AUTS2*. In particular, the differentially expressed genes (DEGs) identified in this study showed extensive overlap with the DEGs found in the Mariani study, including the GABAergic interneuron development-related genes [85]. These studies suggested that abnormal development of GABAergic neuron is one of the common pathological mechanisms of ASD. *RAB39b* is an X-linked gene and codes for a member of the RAS-like GTPase superfamily. LOF mutations of *RAB39b* lead to ASD [86]. hPDBOs carrying the *RAB39b* mutation showed enlarged sizes due to the overproliferation and impaired differentiation of NPCs, further studies revealed *RAB39b* mutation promotes PI3K-AKT-mTOR activity and alters cortical neurogenesis, leading to macrocephaly and autistic-like behaviors [87]. Recently, hPSC-based thalamic organoids and cortex-thalamus assembloids have been developed in vitro, which may hold great promises to do ASD modeling and drug screening in depth [35].

## BOs for modeling neurodegenerative disorders

Neurodegenerative diseases affect millions of people worldwide. Parkinson’s disease (PD), Alzheimer’s disease (AD), and Huntington’s disease (HD) are typical neurodegenerative diseases caused by progressive death of neurons in brain. Despite the neurodegenerative diseases have been widely studied using various of animal models, they failed to fully recapitulate all the disease features in human [88–90]. In this section, we summarize recent findings using hPDBOs for neurodegenerative disease modeling and drug screening.

### Modeling AD with BOs

AD is an age-related neurodegenerative disease, and clinically manifestated with progressive memory loss and cognitive dysfunction [91]. The main pathological features of AD patients were accumulation of amyloid β-peptide (Aβ) peptides and hyperphosphorylated Tau in brain, which will induce the formation of senile plaques and neurofibrillary tangles, and eventually the neuronal cell loss [92]. Previous studies have repetitively shown the existing models, including the monolayer cell culture system and transgenic animal models, were unable to fully recapitulate all of the disease features, due to lack of brain tissue organization and existing of species differences [93]. Even more, the drugs developed from the AD mouse models have failed to demonstrate efficacy in human patients [89]. Thus, developing a more effective and predictable model is a top priority for AD modeling and treatment. hPDBOs, either from AD patient or carrying the AD patient-related mutations, have spontaneously developed the AD pathological features, including accumulation of Aβ aggregates and hyperphosphorylated Tau [94–96]. In 2018, Gonzalez et al. firstly showed familial AD patient iPSC-derived BOs developed the two major pathological features of AD and demonstrated the BOs may represent an alternative model for studying the pathology of genetic forms of AD [95]. Subsequently, Zhao et al. showed BOs from AD patients carrying *APOE4* increased levels of Aβ and phosphorylated Tau, and exacerbated neuronal cell apoptosis and synapse loss, confirming that APOE4-mediated degenerative pathways contributing to AD pathogenesis [97]. Additionally, the BO model also provided a platform for identification of new AD-risk genes. José Pérez et al. firstly revealed that *PITRM1* knockout iPSC-derived BOs spontaneously developed pathological features of AD and revealed PITRM1 played as a protective role in AD pathogenesis [98]. BACE2 is a homologue of BACE1, however, its expression and function in brain is still confusing [99–101]. Using the hPDBO model, Alić et al. have proved that BACE2 is a dose-sensitive AD-suppressor gene in human brain [102]. Most recently, Park et al. have developed a network-based drug-screening platform by integrating mathematical modeling and hPDBOs with the AD pathological features. Using this platform, they have identified several FDA-approved drugs as candidates for curing AD [103]. Taken together, these studies have demonstrated that hPDBOs indeed represent a more ideal and efficient model for AD modeling and drug screening.

### Modeling PD with BOs

PD is the second most frequent neurodegenerative disorder after AD, and characterized by its motor symptoms and the frequent co-occurrence of cognitive and psychiatric symptoms. The prominent pathological changes of PD are degeneration and death of dopaminergic neurons (DANs) in substantia nigra of human midbrain [104]. Due to lack of representative experimental models that can recapitulate the complex multifactorial neurological diseases, the progress in understanding the molecular mechanisms of PD and discovering the disease-modifying treatments is slow. hPDBO models, in particular the midbrain-specific BOs (MOs), offer new possibilities to overcome this issue [21, 105–108]. In 2016, Qian et al. developed the MOs from hPSC using a miniaturized spinning bioreactor, which recapitulated the key dynamic features of developing human midbrain at the molecular, cellular, and structural level. For example, the MOs contained the ventricular zone (OTX2/FOXA2^+^), as well as intermediate (LMX1A/NURR1^+^) and mantle layers (MAP2/TH^+^) neurons [21, 29]. In addition, several pan-mDA neural markers, including the dopamine transporter and DOPA decarboxylase enzyme have been consistently observed in hMOs [108]. Using this model, *LRRK2*^*G2019S*^-associated hMOs have been developed [109], and which have recapitulated the PD-relevant phenotypes, including reduced the numbers of mature DANs and neurite length in comparison to the control organoids. Furthermore, treatment of the *LRRK2*^*G2019S*^ mutant organoids with LRRK2 inhibitor partially rescued the DANs-specific gene expression [110, 111]. These studies showed hMOs may represent an efficient and reliable platform for PD modeling and drug screening in the future.

### Modeling HD with BOs

HD is an autosomal dominant neurodegenerative disease and characterized by motor dysfunction, progressive cognitive deterioration and the behavioral prodrome [112]. it is caused by abnormal expansion of CAG repeat in Huntingtin gene (*HTT*), more than 40 of CAG repeats will confer HD and induce the neural cell death in the striatum and cortex [113]. Previous study has demonstrated BOs-derived from the HD iPSCs with increasing CAG expansions caused the failure of neuroectodermal acquisition, disruption of neural rosette and organoid cytoarchitecture, which could be rescued by knockdown of *HTT* or pharmacologic inhibition of ADAM10 activity, indicating these phenotypes are dependent on CAG-expansion [114]. However, whether the BO models used here correctly mimic what happens in HD patients still need to be investigated. Recently, Andersen et al. have developed a three-part assembloids, consisting of BOs, spinal cord and skeletal muscle spheroids, which can be maintained functionally and morphologically intact for up to 10 weeks in vitro, and may represent a resource for uncovering mechanistic insights into HD and investigating new HD treatment strategies [40].

## BOs for modeling tumor initiation, progression, and invasion

The complexity and heterogeneity of cancers, lead to the variations in the curative effects from person to person, which is an enormous challenge faced in tumor therapy. Developing an experimental model that accurately recapitulates cancer is crucial for the study of cancer biology and development of therapeutic treatments. Traditional in vitro culture models, including 2D human cancer cell or 3D tumor spheroid cultures, are most likely to acquire additional mutations during amplification process due to the genomic instability; while transgenic animal models cannot fully reflect the genetic characteristics of human cancers; patient-derived xenotransplantation models can retain the important features of the primary tumors, however, they are not suitable for high-throughput drug screening owing to the high cost and low implantation efficiency. hPSC-derived 3D organoids are easily accessible and handled, and have more closely recapitulated the relative complicated cytoarchitecture and microenvironment of human organs. Therefore, many of them have been generated to study tumorigenesis and personalized therapy, including glioblastoma (GBM), pancreatic ductal adenocarcinoma (PDAC), lung adenocarcinomas, retinoblastoma, and colorectal cancer organoids [115–120]. Using this model, the researchers have uncovered new mechanistic insights into the tumor development, which are difficult to be learned from other models. For example, PDACs can develop from both acini and ducts, and acinar cell-derived PDACs frequently traverse to pancreatic intraepithelial neoplasia, while PDACs developed from ductal cells are more likely to progress to aggressive cancers [121, 122]. However, how does the cell type of origin affect human PDAC biology and progression is unknown. For this issue, one of the challenges is human acinar cell cultures are short lived and easy to undergo trans-differentiation by acinar-to-ductal metaplasia, while the mouse model is not in a human cell context. Recently, two separate groups from Huang et al. and Breunig et al. have generated the hPSC-based pancreatic duct-like and acinus-like organoids, which have recapitulated the properties of neonatal exocrine pancreas. Using these models introducing the PDAC-associated oncogene mutations, they revealed that *GNAS*^*R201C*^ and *KRAS*^*G12D*^ have lineage-dependent effects on PDAC formation in vitro and in vivo, *GNAS*^*R201C*^ mutated in ductal organoids induced cystic growth more effectively than acinar organoids, whereas *KRAS*^*G12D*^ expressed in acinar organoids is more effective to induce acinar-to-ductal metaplasia-like changes and model PDAC in vivo [119, 120]. This study highlighted the advantages of hPSC-derived organoids in modeling tumor progression of heterogeneity. GBM is the most common intracranial tumor with rapid growth, strong invasion, and easy recurrence. The prognosis of GBM with the highest malignant degree is extremely poor, and the median survival time is less than 15 months [123]. Currently, the main treatment strategies for GBM are by surgery combined with postoperative radiotherapy and chemotherapy. Targeted treatment and immunotherapy strategies are very limited, owing to the presence of blood–brain barrier (BBB) and lack of knowledge mechanism underlying the tumor initiation and progression [124, 125]. Although many models have been developed for preclinical modeling of GBM, their contributions to clinical treatment are very limited due to its molecular heterogeneity and complicated cytoarchitecture and microenvironment of human brain tissue [126, 127]. In this part, we will summarize the recent applications of BOs during the study of GBM initiation, progression, and invasion.

### BOs for modeling GBM initiation and progression

In the last 3 years, hPDBOs have been widely used to model the initiation and progression of GBM [128, 129]. For example, in 2018, by combining with genome-editing technology, hPDBOs have been successfully induced to grow GBM in vitro, which is also called neoplastic cerebral organoids (neoCORs). Using this model, Bian et al. identified that overexpression of oncogene *MYC* or introducing other commonly found gene aberrations from patient GBM into wild type BOs, can strikingly induce tumor overgrowth, which have exhibited many features of cancer, like in vivo expansion and invasion capabilities [116]. More interestingly, they found the induced neoCORs by overexpressing *MYC* showed histopathological features, cellular identities, and transcriptome signatures very similar to those described for human central nervous system primitive neuroectodermal tumor (CNS-PNET), for which no successful animal or in vitro model exists so far [130]. More importantly, *MYC*^*OE*^ alone could initiate CNS-PNET-like neoplasm in BOs within a very short period, while normally it requires additional genetic events such as loss of *p53* and much longer time in animal models, with low incidence [131]. Using the similar strategy, Ogawa et al. have shown activation of oncogene *HRas*^*G12V*^ and simultaneous disruption of *TP53* can induce mesenchymal-like GBM in organoids, and the cells isolated from these organoids can be orthotopically xenografted into immunodeficient mice and exhibited an invasive phenotype. Transcriptome analysis of these organoid-generated putative tumor cells showed gene expression profiles consistent with mesenchymal subtype human GBM [132]. Taken together, these studies demonstrated that neoCORs can be used to model the initiation of different types of GBMs with specific DNA aberrations, including the highly rare and malignant tumors.

### BOs for modeling GBM invasion

GBM is more prone to propagate and ultimately kill patients through diffuse invasion and infiltration into surrounding normal cerebral tissue, instead of by metastasis to other tissues. Thus, 2D cultures and traditional 3D tumor organoids cannot model this critically important cell–cell interactions and the tumor microenvironment, while the other animal models exist marked interspecies differences. hPDBOs cocultured with GBM spheroid or patient-derived glioblastoma stem cells (GSCs) can form glioma BOs to study tumor invasion. Da silva et al. firstly demonstrated that GBM spheroids can spontaneously infiltrate early-stage BOs to form hybrid organoids, which provides a basis for modeling and quantification of the GBM infiltration process [133]. Subsequently, Linkous et al. developed a cocultured system with patient-derived GSCs and hPDBOs, and called their model as GLICO (GLIoma cerebral organoids). They showed the GSCs can deeply invade and proliferate in human BOs, which has phenocopied the patient GBM behaviors and provided a system for modeling tumor cell invasion in a human brain environment [134, 135]. More importantly, using this GLICO model, they demonstrated that GSCs grown within the microenvironment of cerebral organoids were more resistant to drug and radiation-induced genotoxic stress, as seen in GBM patient treatment, such an effect is not recapitulated using standard 2D culture system. These studies demonstrated that GLICO provided a powerful tool for investigating GBM biology in a primitive human brain environment and modeling diverse therapeutic interventions.

## Challenges for existing BO models

**Fig. 2:**
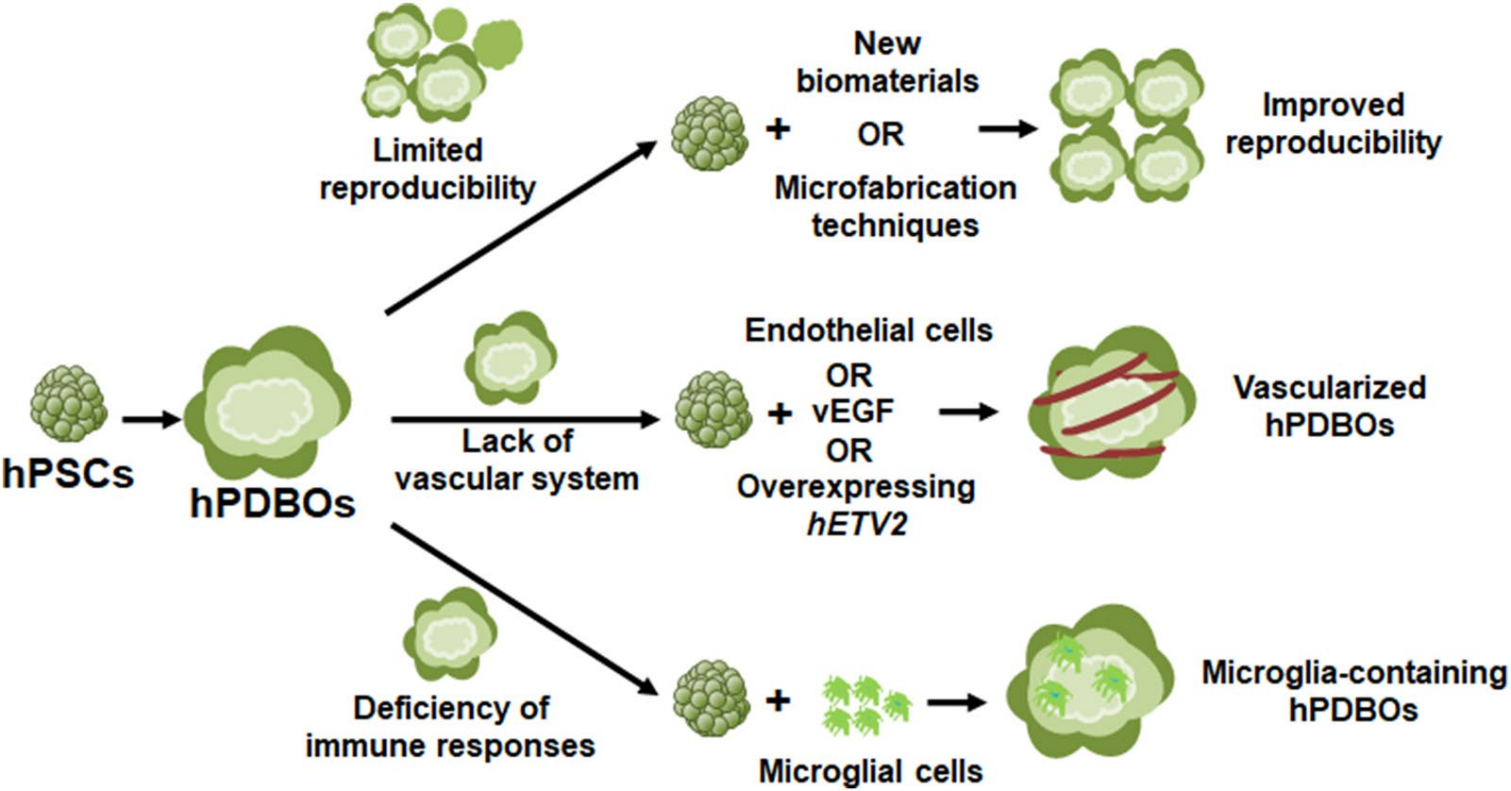
Challenges of existing hPDBOs: 1. Limited reproducibility: attempt to develop homogenous hPDBOs by introducing new biomaterials or combining with microfabrication techniques; 2. Lack of vascular system: different attempts to realize organoid vascularization, including hPDBOs co-culture with endothelial cells, or co-differentiation with VEGF administration or co-overexpression with hETV2; 3. Deficiency of immune responses: attempt to develop microglia-containing brain organoids by co-culture of hPDBOs with microglial cells

In the past decade, numerous of breakthroughs have been made in BO technologies, including the organoid development methodologies and their applications in disease modeling and drug screening. However, this field is still in its infancy, and many of the challenges are existed (Fig. 2).

### Limited reproducibility

One of the major limitations of BOs is their variability within and between organoid batches, which resulted in limited reproducibility and sometimes the data misinterpretation for disease modeling. For example, even in the same batch, BOs generated by unguided protocols showed lots of variations, including the morphologies, sizes and cytoarchitectures [11, 19], resulted from inconsistent neural induction efficiency and matrigel supports. To overcome this issue, Lancaster et al. have used poly (lactide-co-glycolide) copolymer fiber microfilaments as a floating scaffold to generate elongated EBs, by which the resulting organoids displayed improved reproducibility of neural induction and subsequent cortical development [136]. In addition, compared with above-mentioned unguided protocols, cortical organoid generated by guided protocols can achieve reproducible generation with a rich diversity of cell types appropriate for the human cerebral cortex [14, 137]. Recently, Zhu et al. have developed a simple protocol to enable in situ formation of massive BOs from iPSCs on a micropillar array without tedious manual procedures. The optimized micropillar configurations allow for controlled EB formation, neural induction and differentiation, and generation of functional human BOs in 3D culture on a single device. By combining microfabrication techniques with stem cells and developmental biology principles, the proposed method can greatly simplify BO formation protocols and overcome the potential limitations of cell contamination, lower throughput and variance of organoid morphology [138]. Overall, during organoid generation, uniform size, uniform morphology, and synchronized differentiation of EBs should be considered in the future for improve the reproducibility.

### Lack of a functional vascular system

The majority of BOs generated using current protocols resemble an early stage of fetal brain [19, 20, 25, 26, 139], and which are not suitable to investigate the physiological and pathological aspects of adult brain, like the late-onset neurodegenerative diseases. The major reason is the BOs lack of a circulation system with blood vessels to continually supply of oxygen and nutrients for extended in vitro culture. To overcome this issue, several strategies have been explored to realize the BO vascularization. BOs co-cultured with endothelial cells or co-differentiated with endothelial-like cells (supplementation of VEGF or overexpression of *hETV2*) have induced the formation of vascular-like system without reducing neural morphogenesis [140–142]. More recently, Shi et al. have generated the vascularized BO model (vOrganoids) consisting of typical human cortical cell types and a vascular structure for over 200 days, scRNA-seq analysis illustrated that DEGs of vOrganoids are related to blood vessel morphogenesis. Transplantation of vOrganoids into mouse cortex resulted in the construction of functional human-mouse blood vessels that promoted cell survival in the grafts [143]. In addition, some models have incorporated primary astrocytes, hiPSC-derived pericytes with endothelial cells, astrocytes, and neurons to model the BBB. These BBB models show in vivo characteristics with high transendothelial electrical resistance and expression of tight junction proteins [144–147]. Despite these advances, the BBB platform and differentiation protocols still require optimization to develop functional vasculature in BOs.

### Deficiency of immune responses

Human brain is composed of various types of cells, including neural cells and non-neural cells, such as microglia. Among of them, neural cells are derived from neuroectoderm, while the other cells, such as microglia is from mesoderm. However, most of the current protocols were used to induce the neuroectodermal fate, and the other lineage cells are largely missing in hPDBOs, which greatly limited its application for modeling brain disorders caused by the interactions between non-neural and neural cells. Microglia, as the resident immune cells of CNS, plays essential roles in the initiation and progression of neurodegeneration diseases and cancers [148–150], therefore, microglia induction in BOs would be valuable to study its functions in these diseases. Ormel et al. have reported that BOs generated without dual-SMAD inhibition innately contain mesodermal progenitors, which can differentiate into mature microglia (Iba1^+^) under the CNS microenvironment [151]. In addition, co-culturing BOs with hPSC-differentiated microglia-like cells showed a certain degree of response to pro-inflammatory stimuli [152, 153]. However, creating an environment to induce microglia in BOs that could exhibit inflammation and immune responses like in human brain remains challenging.

## Conclusion and future perspectives

**Fig. 3:**
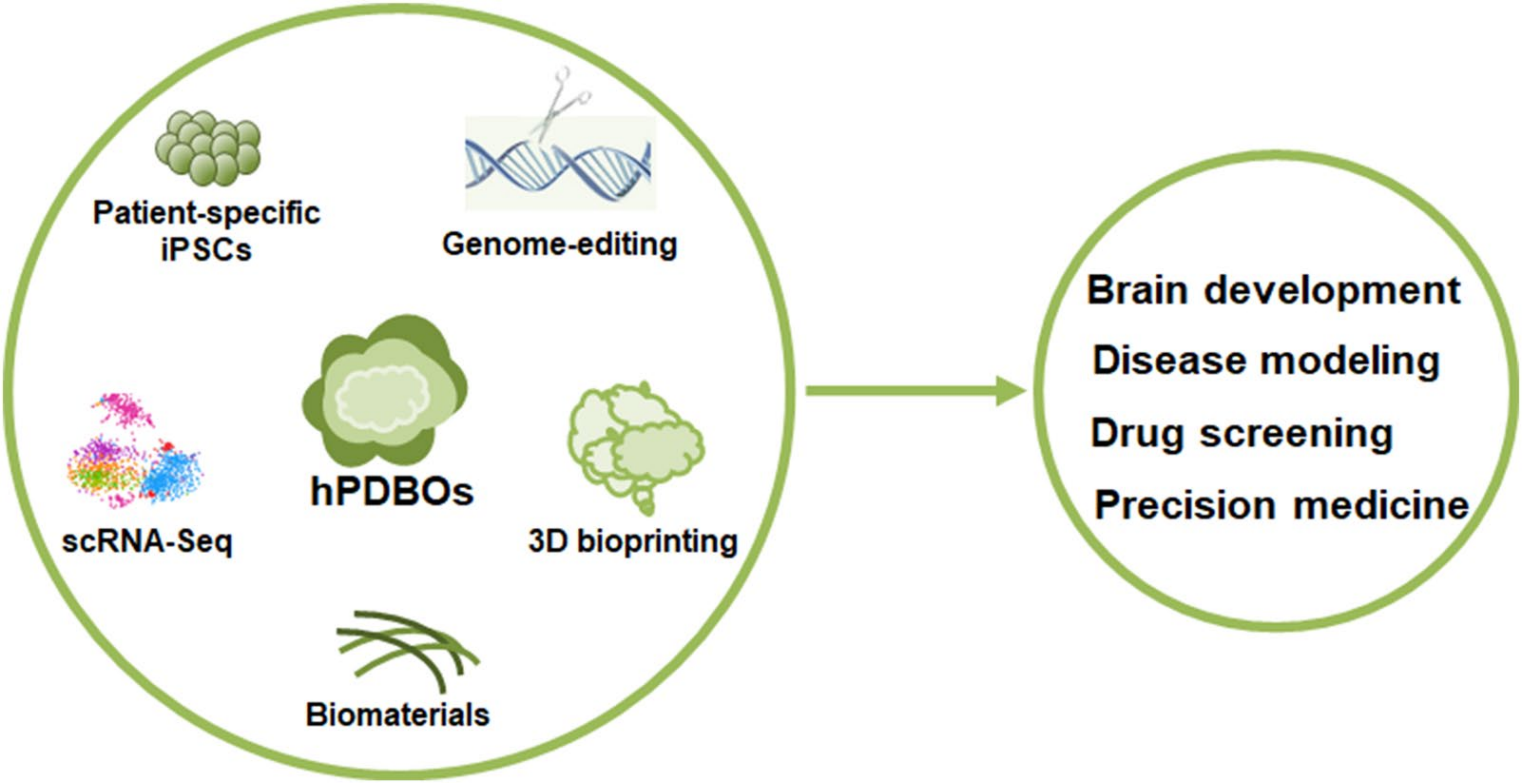
Future perspectives in hPDBOs: The combinations of hPDBOs and innovative technologies, including cell reprogramming, genome-editing, 3D bioprinting, scRNA-seq, and biomaterials will greatly improve the brain organoid system, and which will facilitate us to model human brain development and disorders, and to perform drug discovery and personalized therapeutics in the future

As the only in vitro platform that can recreate the 3D architecture of human brain and recapitulate the process of human neurodevelopment, hPDBOs have made a tremendous breakthrough for modeling brain disorders and cancers. However, it is still with less than a decade of history and in its infancy. Continuous advances of 3D-culture systems and their integrations with innovative technologies, including cell reprogramming, genome-editing, 3D bioprinting, single-cell transcriptomics, and biomaterials (Fig. 3), will lead the BOs to become invaluable models for better understanding of the fundamental biology of brain disorders and cancers in the future.

## Data Availability

Not applicable.

## Abbreviations

3D: Three-dimensional
BOs: Brain organoids
hPSCs: Human pluripotent stem cells
hPDBOs: HPSC-derived BOs
hESCs: Human embryonic stem cells
iPSCs: Induced pluripotent stem cells
EB: Embryoid body
SNO: Sliced neocortical organoid
ALI-CO: Air–liquid interface cerebral organoid
scRNA-seq: Single cell RNA-sequencing
ASD: Autism spectrum disorder
NPCs: Neural progenitor cells
oRGs: Outer radial glia cells
PCM: Pericentriolar material protein
ZIKV: Zika virus
TSC: Tuberous sclerosis complex
TS: Timothy syndrome
hCS: Human cortical spheroids
hSS: Human subpallium organoids
DEGs: Differentially expressed genes
PD: Parkinson’s disease
AD: Alzheimer’s disease
HD: Huntington’s disease
Aβ: Amyloid β-peptide
DANs: Dopaminergic neurons
MOs: Midbrain-specific brain organoids
HTT: Huntingtin gene
GBM: Glioblastoma
PDAC: Pancreatic ductal adenocarcinoma
neoCOR: GBM organoid neoplastic cerebral organoid
CNS-PNET: Central nervous system primitive neuroectodermal tumor
GSC: Glioblastoma stem cells
vOrganoid: Vascularized brain organoid model
GLICO: GLIoma cerebral organoids
BBB: Blood–brain barrier
CNS: Central nervous system
